# Metabolic Perturbations of Kidney and Spleen in Murine Cerebral Malaria: ^1^H NMR-Based Metabolomic Study

**DOI:** 10.1371/journal.pone.0073113

**Published:** 2013-09-06

**Authors:** Soumita Ghosh, Arjun Sengupta, Shobhona Sharma, Haripalsingh M. Sonawat

**Affiliations:** 1 Department of Chemical Sciences, Tata Institute of Fundamental Research, Mumbai, India; 2 Department of Biological Sciences, Tata Institute of Fundamental Research, Mumbai, India; Instituto de Investigación Sanitaria INCLIVA, Spain

## Abstract

A significant fraction of global population is under the threat of malaria. Majority of annual death is due to the more complicated form of the infection i.e. the cerebral form, also known as Cerebral Malaria (CM). Host parasite interaction is known to cause a cascade of events in various tissues like brain, liver, kidney, and spleen. We have employed ^1^H NMR based metabolomics to understand the specific perturbations of various tissues in CM. In our previous paper we have delineated the differences between CM vis-a-vis non-cerebral malaria (NCM) mice in serum, liver and brain. In this paper we focus on their differences of metabolic profile in kidney and spleen as kidney dysfunction and splenomegaly are known to be associated to neurological outcome of the disease. Moreover we have also looked into how the biological compartments (kidney, spleen and serum) interact with each other. The various metabolites involved in such interactions and their correlational aspects across the compartments have been studied in CM, NCM and control mice. The idea was to find out the specific pathways that are altered in CM mice. Our results demonstrate that both the kidney as well as spleen metabolism are differentially perturbed in CM with respect to NCM. The results point out that glutamate levels are decreased in CM mice with respect to NCM mice both in case of spleen and kidney while creatine, *myo*-inositol and betaine levels are increased in kidney of CM mice with respect to NCM mice. From the analysis of Multiway Principal Component Analysis (MPCA) we see that lipid metabolism and TCA cycle is altered in kidney and spleen.

## Introduction

Cerebral malaria (CM) is one of the complicated forms of malaria and the neurological outcome of this disease is often correlated to the dysfunction of liver [1], brain [2], [3], [4], acute renal failure [5], [6], [7] and splenomegaly [8], [9], [10]. These are some of its serious complications which could be life threatening. These features are many fold higher in *Plasmodium falciparum* infected patients, and are rarely reported in patients infected with *P. vivax*. The complication occurs frequently in non-immune adults and older children infected with *P. falciparum* [11]. In our previous study we had reported on metabolic changes in brain and liver in the host in the murine model of the disease. Here we focus on the alterations in spleen and kidney.

The fatality linked with CM increases to ∼50% when the disease co-occurs with renal failure and metabolic acidosis. In several instances CM is associated with renal dysfunction [1], [6], [12], [13]. Splenomegaly, on the other hand, is due to protection or background immunity or other causes such as hemoglobinopathies. However, splenomegaly is thought to have a causal relationship with CM not only in humans but in animal models as well [9], [14], [15]. Involvement of spleen in splenomegaly in malaria renders this organ to rupture [16], [17]. In addition, sequestering of infected RBCs in kidney and spleen might alter the metabolism in the host [18]. Renal ischemia is known to result in tubular necrosis which, in turn, leads to renal failure [19]. However the exact mechanism of renal failure is not completely understood [18]. We believe that kidney dysfunction and splenomegaly causes the perturbation of metabolism in these specific tissues and, in turn, also impacts on overall metabolism.

Using ^1^H NMR spectroscopy and multivariate analyses we had investigated metabolic alterations in liver, serum and brain of mice infected with *P. berghei* that exhibited symptoms of cerebral malaria [4]. We had observed differential metabolic changes in these tissues in mice with CM when compared with those with non-cerebral malaria (NCM). Here we present the data from our experiments on NMR spectroscopy of kidney and spleen of malaria parasite infected mice that had transited into CM vis-à-vis those that remained with NCM. We observe fingerprints of kidney and spleen that are specific for CM and NCM. We further report here on the correlational aspect of the various metabolites from these tissues with serum. We find metabolic correlates in spleen and kidney of CM while keeping NCM of same genetic background. The results obtained in this study point towards perturbation in lipid metabolism and TCA cycle in CM. We believe this will shed light on the metabolic events in specific tissues and the overall status of the host as well as improve our understanding of the role of these tissues in maintaining homeostasis during CM.

## Methods

### Animal Handling

The study was approved by the Animal Ethics Committee of the Tata Institute of Fundamental Research (IEAC approval no: TIFR/IAEC/2010-3). The animals used in these experiments were treated as per the guidelines of the Animal Ethics Committee. 26 female C57BL/6 mice, 6–8 weeks old and weighing 20–25 g were used for the study. The animals were maintained in 12 hours day and night cycle. They had free access to water and standard food pellets. The temperature was maintained at 22±2°C. Out of 26 mice, 21 mice were inoculated with 10^7^ iRBCs with *P. berghei* and 5 of them were kept as controls. Rectal temperature of all the mice was measured daily using a digital thermometer. Mice were considered to have CM if they had rectal temperature <34°C [20]. Other neurological symptoms such as ataxia, convulsions, coma [21] were also noted for all the infected mice. The NCM mice, on the other hand, did not show any neurological symptoms and had body temperature >34°C. Thus categorization of animals as having CM or NCM was unambiguous. A total of 4 mice died during the experiment and were excluded from the study. On day 9, 17 infected (cerebral and non cerebral mice) were sacrificed along with 5 control animals.

### Sample Preparations

Blood was collected (∼100 µL) by retro-orbital bleeding. The mice were then sacrificed by cervical dislocation [22], [23]. This was immediately followed by dissection of the organs. The blood samples were incubated at 37°C for 10 mins and centrifuged for 10 mins at 13100 g at 4°C. The supernatant was collected, frozen immediately in liquid N_2_ and stored at −80°C. For NMR experiments 50 µl of serum was mixed with 450 µL of buffer (0.075M Na_2_HPO_4_.7H_2_0. 4% NaN_3_, 0.02% TSP, pH 7.4) and 50 µL of D_2_O. The buffer recipe was provided by Bruker Biospin, Metabonomic unit. The pH of the samples was checked before experiments.

### Preparation of Tissue Extract

After sacrificing the animal, the kidneys and spleens were quickly excised out, snap frozen in liquid N_2_ and stored at −80°C till further processing. Each whole kidney and whole spleen was weighed and transferred to a glass homogenizer. The tissue was homogenized with ice cold methanol (4 ml/g wet weight) and ice-cold water (0.85 ml/g wet weight). To it further 2 ml/g of chloroform was added and vortexed. This was followed by further addition of 2 ml/g of water and chloroform successively and vortexed, allowed to rest for 15 minutes on ice and centrifuged at 1000 g [24]. The supernatant was dried in a vacuum concentrator and stored at −20°C until further use for NMR experiments. The dried mass was reconstituted in D_2_O containing 0.02% TSP (3-(trimethylsilyl)-2, 2′, 3, 3′-tetradeuteropropionic acid for NMR spectra acquisition. The samples were used for NMR experiments.

### ^1^H NMR of Sera

^1^H NMR spectra of sera were acquired at 310 K on AVANCE 700 MHz Bruker spectrometer equipped with triple resonance probe. The pulse sequence used for this experiment was of the form -RD-90°-(τ-180-τ-)_n_-90°-ACQ. Here (τ-180-τ-)_n_ is a CPMG block which helps attenuate broad signals from macromolecules. A relaxation delay (RD) of 4 s was used between consecutive pulses [24]. A loop count (n) of 128 and τ of 300 µs, which is half of the inter-pulse delay between two successive 180°, was used. In these experiments 32 transients were collected into 88640 data points using a spectral width of 20.06 ppm. The 90° pulse length was determined carefully for each sample. The FIDs were subjected to an exponential multiplication leading to an additional line broadening of 1 Hz, a Gaussian multiplication of 0.01 Hz, and a sine squared bell apodization function prior to Fourier transformation. The resulting spectra were phased and baseline corrected by the AU program provided by Bruker Biospin. The assignments of metabolites were based on 2D-COSY [25], 2D-(^1^H-^1^H) TOCSY [26] and 2D J-resolved spectroscopy [27] experiments. The assignments were further confirmed using Human Metabolome Databases (HMDB) and were also cross checked from literature [28]. For COSY and TOCSY experiments, 64 transients per increment and 256 increments were collected in the indirect dimension. A sine squared bell function with 2048 and 1024 digital points were used for processing. Exponential multiplication of 0.20 and 0.30 Hz in the direct and indirect dimension was used respectively. TOCSY was processed using SINE function. Exponential multiplication of 0.20 and 0.30 Hz in the direct and indirect dimension was used respectively. In addition, Gaussian multiplication of 0.1 Hz in the indirect dimension was applied for the TOCSY.

### ^1^H NMR of Spleen and Kidney

^1^H NMR spectra were acquired for all the extract samples of kidney and spleen on AVANCE 700 MHz Bruker spectrometer at 300K. The pulse sequence used for the experiment was 1D NOESY presat (NOESYPR1D); the pulse sequence of which is of the form RD-90-t1-90-t_m_-90-ACQ [24]. The relaxation delay (RD) of 4 s was used for all the experiments. The spectral width of 20.06 ppm was used. The number of transients of 32 and the time domain data points of 32000 was used. In all the experiments the 90° pulse lengths were determined individually. The baseline and the phase correction of the spectra were done with the help of AU program provided by Bruker Biospin.

### Data Pre-processing

The ^1^H NMR spectra obtained after phase and baseline corrections were subjected to multivariate statistical processing. Spectral region of 0.5 to 4.15 ppm was bucketed into frequency window of 0.003 ppm. The region corresponding to water (4.15 to 5.5) ppm was excluded to avoid possible artifacts due to pre-saturation of water. Furthermore, the aromatic region, which had poorer signal to noise ratio, in comparison to the aliphatic region, was analyzed separately. The bins were integrated and the resulting integrals were normalized to the working region (0.50 to 4.15 ppm) of the spectrum in order to correct for inter-sample variation in dilution. All the binning and the normalizations of the data matrix were performed using AMIX 3.8 (Bruker). The data matrix obtained was imported into SIMCA P+ 12.0 (Umetrics AB, Sweden) for further multivariate analyses. Specific resonances in the aromatic region were integrated using TOPSPIN 2.0 and analyzed using univariate analyses.

## Statistical Analyses of the Data

### Multivariate Data Analyses – PCA and OPLS-DA

The 2D data matrix obtained from AMIX was subjected to multivariate statistical analyses. The 2D data matrix was pareto scaled and then subjected to PCA and OPLS-DA. The first step of multivariate analyses was Principal Component Analysis (PCA) which is an unsupervised method. PCA was done to see if there is a trend in the dataset and also to find out if there are any outliers present. PCA was followed by Orthogonal Projection to Latent Structures (OPLS-DA) [29] which is a supervised method and gives segregation between two specified classes along the predictive component. A total of six such models were prepared (CM vs. NCM, NCM vs. Control and CM vs. Control) for two tissues; spleen and kidney. The two parameters namely the R^2^(cum) and Q^2^Y(cum) judge the OPLS-DA model. While R^2^ (cum) explains the total variation, Q^2^Y is a cross validation parameter which indicates the predictability of OPLS-DA. In each cross validated round 1/7th of the data were taken out. The scores are visualized as 2D scores plot. The difference in the relative concentration of metabolites in the sample are interpreted by ‘S’ plot which gives the loading as well as the correlation values of the chemical shifts of metabolites related to the class segregation denoted by p[1] and p(corr) respectively. This analysis is aided by the VIP plot which reflects the importance of the chemical shifts both with respect to class segregation and the loading (p[1]). The difference in the relative concentrations of metabolites in kidney and spleen of two specified classes are interpreted by OPLS coefficient plot [30]. This plot was generated using a script developed in-house in GNUPLOT 4.4. In this plot OPLS modeled covariance, [cov (t_p_, X)], is plotted against the chemical shift and is colored with OPLS modeled correlation [cor (t_p_, X)]. The orientation of the peaks signifies relative concentration of metabolite in serum/specific tissues (spleen or kidney) of CM, NCM or controls. A color bar associated to the plot indicates the correlation of the metabolites in segregating between classes. The most correlated metabolite toward segregation in OPLS-DA analysis is further analyzed by univariate analysis.

### Univariate Analysis and Fold Change

In the ^1^H NMR spectra, the peaks representing the potential biomarkers in the specific tissues obtained from OPLS-DA were integrated and normalized to the total spectral intensity using TOPSPIN 2.1. The significance of the levels of the metabolites is checked by non-parametric Kruskal-Walis test of Sigma-Plot 11.0. The fold changes were calculated by taking the ratio of the average of the infected group by the average of the control group. In case where fold change was below 1 it was replaced by the negative value of its inverse.

### Multiway Principal Component Analysis

Multiway Principal Component Analysis (MPCA) is used to understand the metabolic correlations between different organs and compartments of the body [31], [32]. Kidney, spleen and serum constitute three different compartments of the body and MPCA gives metabolic correlations that occur across them. MPCA is an extension of PCA and is used for higher dimension of data matrix. Here the N order data (three in this case) are decomposed into scores (t) loadings (p) and the residual matrix (E). The residuals are considered to the non-deterministic part of the data matrix. MPCA models were prepared, to study the metabolic correlation of kidney, spleen and serum. 3D matrix for MPCA was prepared in Matlab 7.0.1 where the first axis corresponds to spectral variables, the second axis corresponds to compartments and the third corresponds to individual animals. The analysis of the MPCA is done using Solo 6.0 (eigenvector research incorporated software). The peaks in MPCA loading plot were assigned in accordance to the resonances in the ^1^H NMR spectrum of corresponding compartment.

## Results

It is known that C57BL/6 infected with PbA is a model of experimental cerebral malaria [33], [34]. The cumulative incidence of CM is variable and it ranges from 50–100% [35]. Previously we have reported a cumulative incidence of 40–60% in our setup [36]. In this experiment, eight mice transited to the cerebral form of the disease while nine of them remained NCM, four mice were found dead during night and were not considered in the analysis. We used the mice with CM and NCM for the analysis.

### Differential Metabolic Alterations in CM and NCM – Kidney

**Figure 1:**
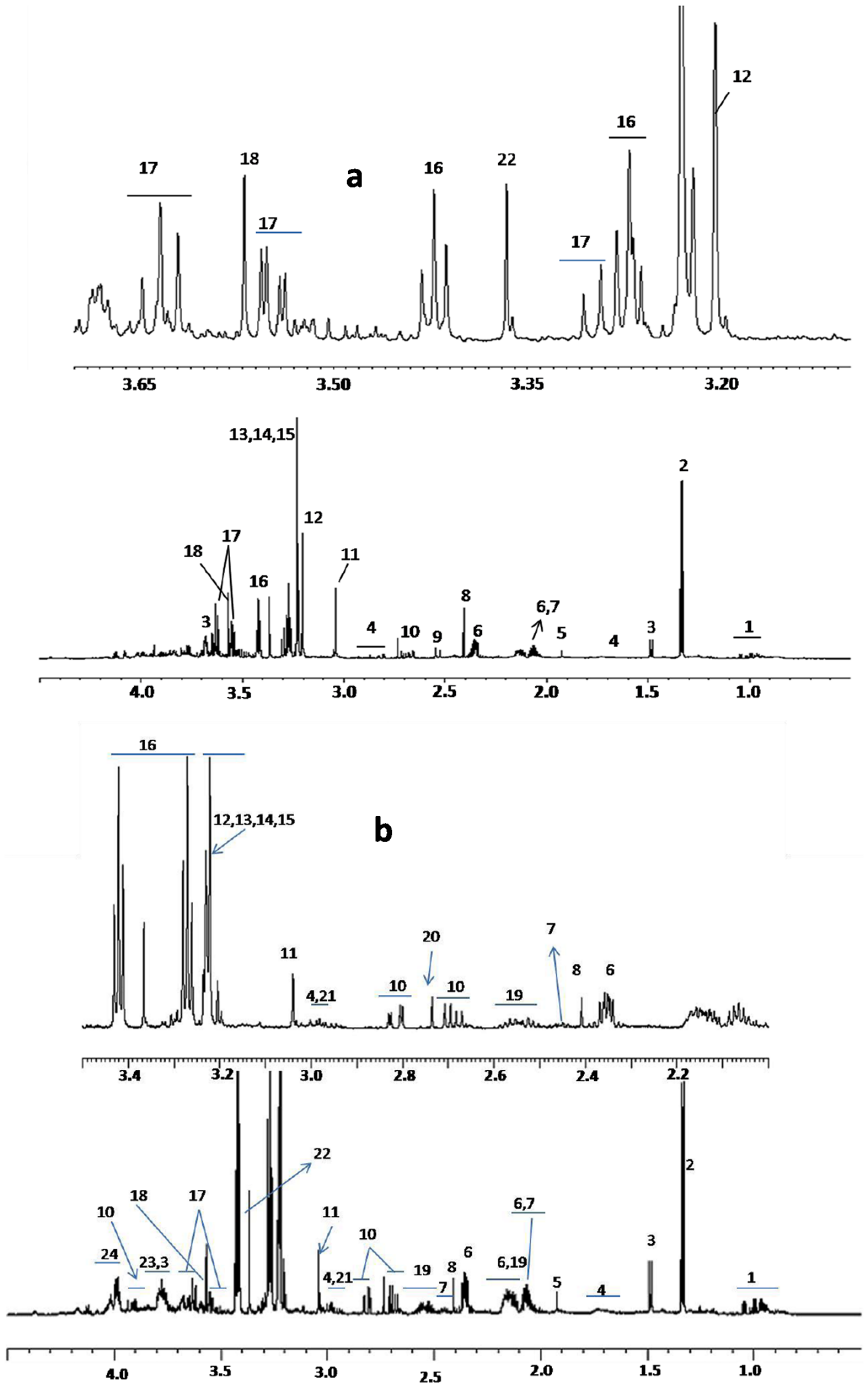
^1^H NMR spectrum of MeOH/Chloroform extract of kidney and spleen. (a) Typical ^1^H NMR spectrum of methanol extract of kidney of control female C57BL/6. (b) Typical ^1^H NMR spectrum of methanol extract of spleen of control female C57BL/6. Keys: 1-BCAA (leucine, valine, isoleucine), 2-lactate, 3-alanine, 4-lysine, 5- acetate, 6-glutamate, 7-glutamine, 8-pyruvate, 9-citrate, 10-aspartic acid, 11-creatine, 12-choline, 13-phosphocholine, 14-glycerophosphocholine, 15-betaine, 16-taurine, 17-*myo*-inositol, 18-glycine, 19-glutathione, 20-sarcosine, 21-histamine, 22-*scyllo*-inositol, 23-ascorbate, 24-*O*-phosphoethanolamine.

**Figure 2:**
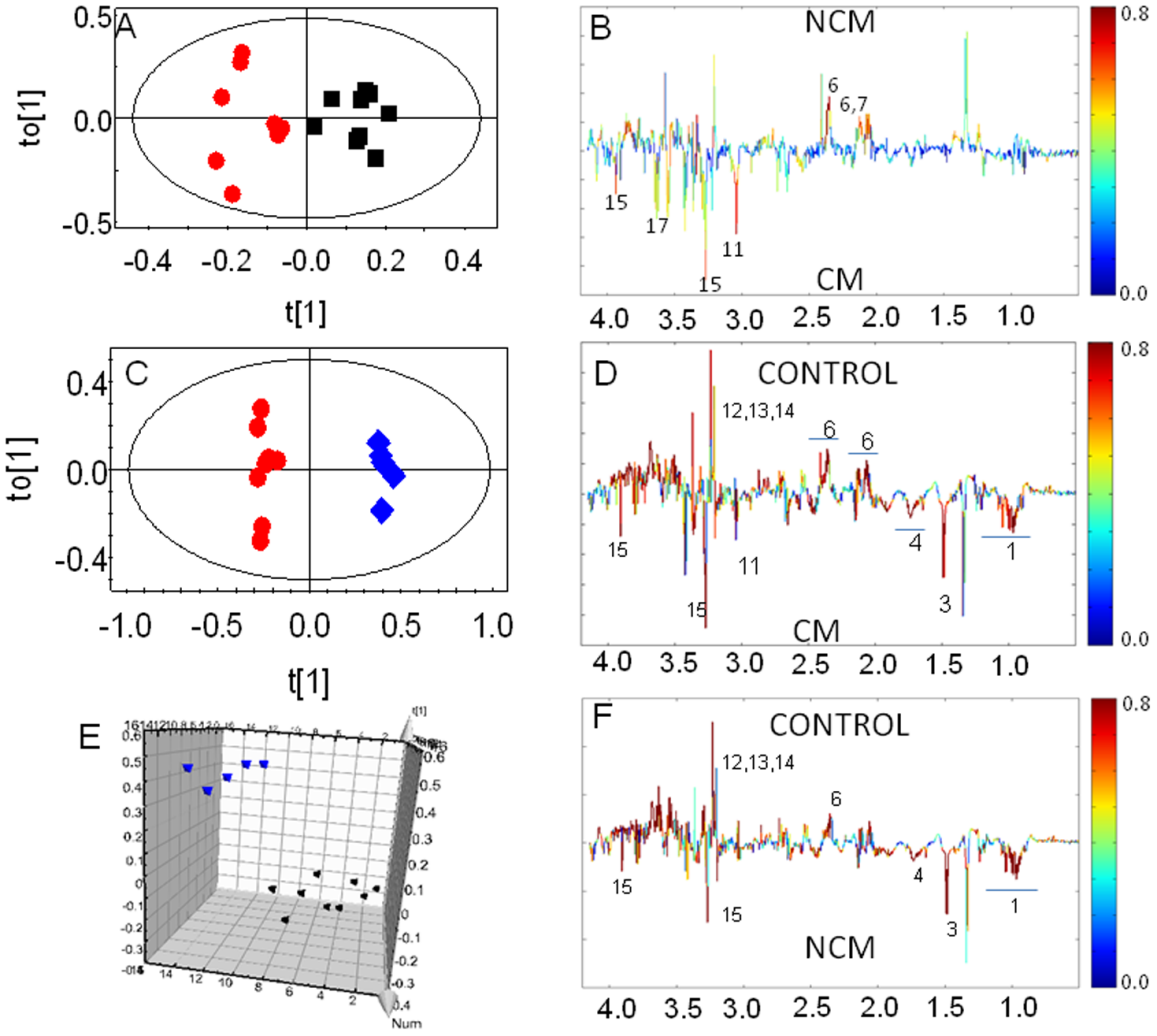
OPLS-DA score plot of kidney extract of CM and NCM and control. (a) OPLS-DA scores plot of CM and NCM. (b) OPLS-DA loadings plot of (a). (c) OPLS-DA scores plot of CM and control. (d) OPLS-DA loadings plot of (c). (e) OPLS-DA scores plot of NCM and control. (f) OPLS-DA loadings plot of (e). OPLS-DA scores plot of metabolite profiles based on ^1^H NMR profile. Each red circles denotes score of one ^1^H NMR of extract of kidney of CM mice and the black circle denotes score of one ^1^H NMR of kidney extract of NCM C57BL/6 mouse at day 9 p.i in an OPLS-DA analysis and blue circles denote score plot of ^1^H NMR of control mouse. The ellipse is a 95% Hotelling T2. The color of the signals in this plot signifies the contribution of the metabolites towards class segregation between kidney of CM NCM C57BL/6. A color bar associated to the plot indicates the correlation of the metabolites in segregating between classes (CM NCM kidney) with least significance of color blue and highest significance of red color. The upward orientation of the peaks denotes relatively higher concentration of the corresponding metabolites in NCM and vice-versa.

The representative ^1^H NMR spectra of kidney and spleen of mice infected with *P. berghei* ANKA (Figure 1), assigned using 2D NMR techniques and literature [37], [38]. This was further aided by (Human Metabolome database, Madison Metabolomics Consortium database). OPLS-DA modeling of the ^1^H NMR spectra of kidney extracts of the control animals with those with that of NCM and CM show distinct clusters (Figure S1a). Three separate pair-wise OPLS-DA models were prepared for three groups, namely CM vs. NCM; CM vs. Control and NCM vs. Control (Figure 2a–f). The respective R^2^X(cum) and Q^2^Y(cum) of the model are listed in Table 1. It is evident from Table 1 that the three groups of animals are distinct from each other as reflected by the high Q^2^ in the OPLS-DA analysis.

**Table 1:** Parameters for pairwise OPLS-DA models for kidney and spleen of CM vs. NCM, NCM vs. control and CM vs. control.

Model	R^2^X	Q^2^Y
CM vs NCM (kidney)	0.37	0.56
CM vs. Control (kidney)	0.70	0.95
NCM vs. Control (kidney)	0.51	0.93
CM vs.NCM (spleen)	0.89	0.76
CM vs. Control (spleen)	0.72	0.95
NCM vs. Control (spleen)	0.72	0.97

**Figure 3:**
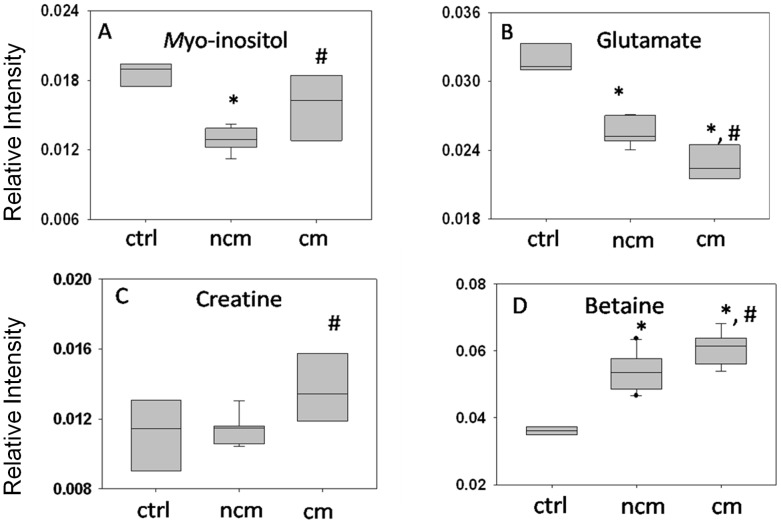
Relative levels of metabolite in kidney: (a) *Myo*-inositol, (b) glutamate (c) creatine and (d) betaine. In (a), (b) and (d) bonferroni test was used because the criteria of normality was fulfilled. In (c) mann whitney test was applied. (*) denote significance of the level with respect to controls while (#) denote significance of the level with respect to NCM.

The OPLS-DA scores plot of ^1^H NMR spectra of CM and NCM are distinct (Figure 2a). Additionally ROC was plotted with the cross validated “Y” which is also known as predicted class [39]. The AUC of the plot is 0.94 (Figure S1b). Figure 2b indicates that glutamate and creatine contribute towards the segregation of the kidney of NCM and CM. The other metabolites like *myo*-inositol are also involved in segregation but have poor correlation towards segregation. It is evident that glutamate concentration is decreased in CM; creatine and betaine concentration is increased in CM, when these groups (CM and NCM) are compared to each other. In order to understand the absolute difference in the levels of metabolites the two infected groups were compared with that of the controls. Figure 2c, 2e and Table 1 indicate that the infected groups are clustered separately with respect to controls. From Figure 2d, 2f it is evident that alanine, branched chain amino acids, lysine and betaine are increased while glutamate is decreased in both CM and NCM (glutamate more so in CM) and *myo*-inositol is decreased in NCM. This is further corroborated in the plot of relative levels of metabolites in kidney (Figure 3) and ‘fold change’ calculation of the metabolites in CM and NCM with respect to controls (Table 2).

**Table 2:** Fold change of metabolites in kidney and spleen of NCM and CM with respect to controls for the metabolites which are significant in the class segregation as appeared in OPLS-DA analysis.

Tissue	Metabolite	Chemical Shift (ppm)	Fold Change (CM)	Fold Change (NCM)
Kidney	Creatine	3.03	1.2	1.0
	Glutamate	2.34, 2.1, 2.04, 3.74	−1.4*	−1.2*
	*Myo*-inositol	3.26, 3.5, 3.6, 4.05	1.2	−1.3*
	Betaine	3.26, 3.9	1.7*	1.4*
Spleen	Glutamate	2.34, 2.1, 2.04, 3.74	−1.4*	−1.2*
	*O*-phoshpoethanolamine	4.02, 3.23	−1.2	−1.4*
	*Scyllo*-inositol	3.35	−1.8	−2.3*

* Significant fold change.

### Differential Metabolic Alterations in CM and NCM – Spleen

**Figure 4:**
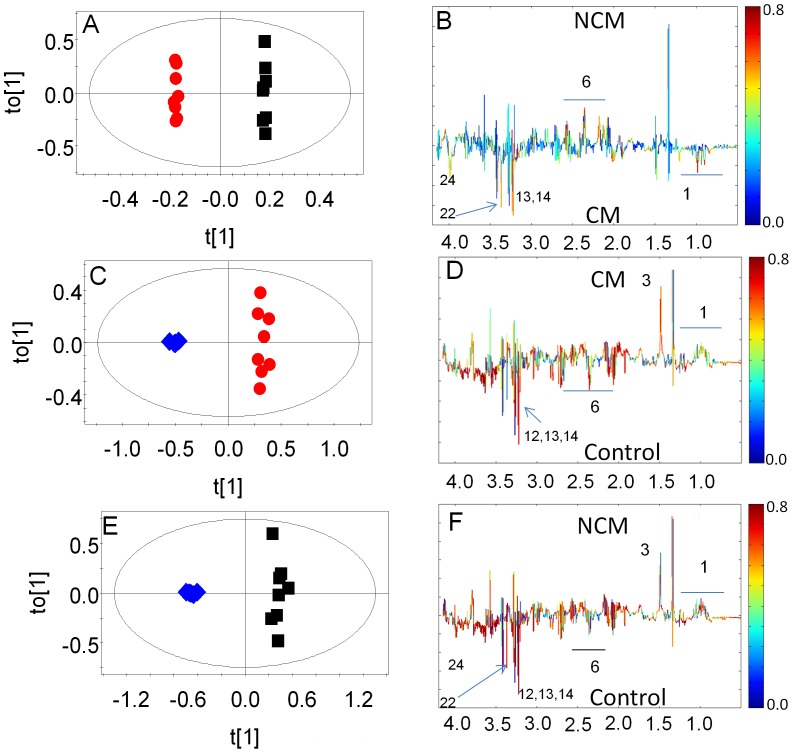
OPLS-DA score plot of spleen extract of CM and NCM and control. (a) OPLS-DA scores plot of CM and NCM. (b) OPLS-DA loadings of (a). (c) OPLS-DA scores plot of CM and control. (d) OPLS-DA loadings plot of (c). (e) OPLS-DA scores plot of NCM and control. (f) OPLS-DA loadings plot of (e). OPLS-DA scores plot of metabolite profiles based on ^1^H NMR profile of spleen. Each red circles denotes score of one ^1^H NMR of extract of spleen of CM mice and the black circle denotes score of one ^1^H NMR of spleen extract of NCM C57BL/6 mouse at day9 p.i. in an OPLS-DA analysis while the blue ones denote the control mice. The ellipse is a 95% Hotelling T2.

**Figure 5:**
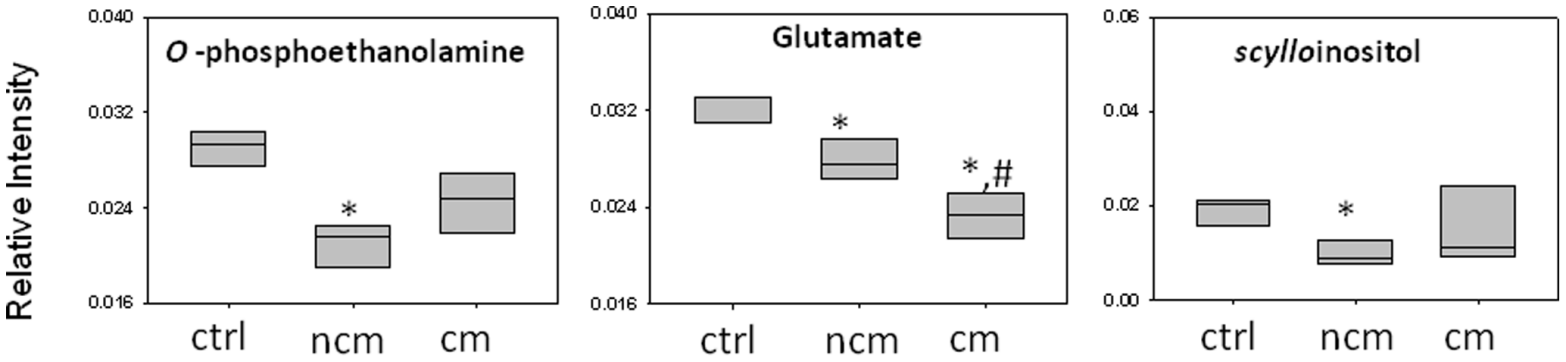
Relative levels of metabolite in spleen: (a) o-phosphoethanolamine, (b) glutamate, (c) *scyllo*-inositol. In (a) and (c) Krushkal-walis test was applied. In (b) bonferroni test was used because the criteria of normality was fulfilled. * Significant with respect to controls while # significant with respect to NCM.

The OPLS-DA model of ^1^H NMR spectra of spleen of mice with CM and NCM and control shows clear segregation (Figure S1c). The pairwise OPLS-DA models created for CM vs. NCM, CM vs. control, NCM vs. control show clear segregation amongst the specified classes (Figure 4a, 4c, 4e and Table 1). R^2^X(cum) and Q^2^Y(cum) of the respective model are listed in Table 1. The ROC of the model of CM vs. NCM was plotted. The AUC of the curve is 1.0 (Figure S1d). The contributing metabolites towards this segregation of CM vs. NCM spleen are relatively low concentration glutamate and high levels of *scyllo*-inositol, cholines, BCAA in CM (Figure 4b). The infected groups cluster distinctly when compared to controls (Figure 4c, 4e and Table 1). Glutamate is decreased in the animals with both CM and NCM with respect to controls (Figure 5). In the model created for infected groups (CM/NCM) and control, nearly all the contributing metabolites have same trend in infected group when compared to controls. Alanine, glutamine, pyruvate, glutathione, glycine are increased while aspartate, choline, phosphocholine, GPC, glutamate and *scyllo*-inositol are downregulated in the infected groups. In addition, *o*-phosphoethanolamine is downregulated in NCM (Table 2). The ‘fold change’ of metabolites listed in Table 2 also clearly indicate that glutamate is decreased in spleen of both CM and NCM with respect to controls (more so in CM). *Scyllo*-inositol and *o*-phosphoethanolamine is decreased in NCM spleen with respect to controls.

The concentration of the most significant metabolites in the analysis of kidney and spleen is listed in Table S1.

### Inter-compartment Correlations

#### CM mice

In order to understand the metabolic correlations among the compartments MPCA models were created for serum, kidney and spleen for CM, NCM and control.

**Figure 6:**
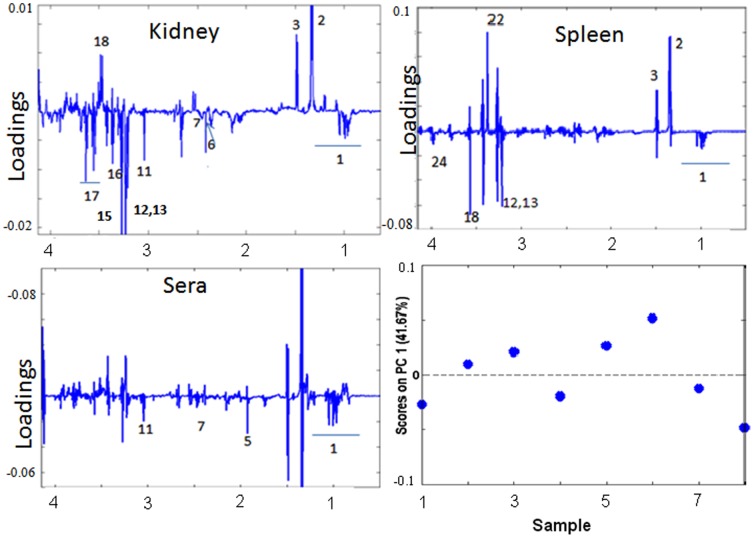
MPCA plot along PC1 of CM animals. (a–c): MPCA loadings along kidney spleen and serum respectively. (d) MPCA scores plot.

Along PC1 decrease in glutamate, glutamine in kidney is associated with decrease of creatine, *myo*-inositol, BCAA and taurine in kidney and increase of lactate and alanine in kidney (Figure 6). Further this decrease is also correlated to increase in *scyllo-*inositol, alanine and lactate in spleen, decrease in phosphoethanolamine, cholines and BCAA, glycines in spleen. Further glutamate in kidney is positively correlated to BCAA, glutamine, acetate in sera (Figure 6). Along PC2 a decrease in glucose and GPC in sera is associated with an increase of alanine in kidney, spleen and serum, as well as increase of lactate in kidney and spleen. (Figure S2). In addition, a decrease of serum glucose is linked to increase of serum lipids and decrease of *o-*phosphoethanolamine in spleen. Serum glucose level is also negatively associated with creatine and positively with *myo*-inositol in kidney. Serum glucose is negatively associated to branched chain amino acids in sera, kidney and spleen. Along PC3, lipids/lipoproteins in serum is anti correlated to lactate in serum and kidney, correlated to alanine in kidney and BCAA in sera and kidney. It is positively correlated to glycine in kidney and spleen. Moreover it is anticorrelated to *scyllo*-inositol, taurine in spleen, *myo*-inositol in kidney, choline in spleen, kidney and serum (Figure S3).

#### NCM mice

**Figure 7:**
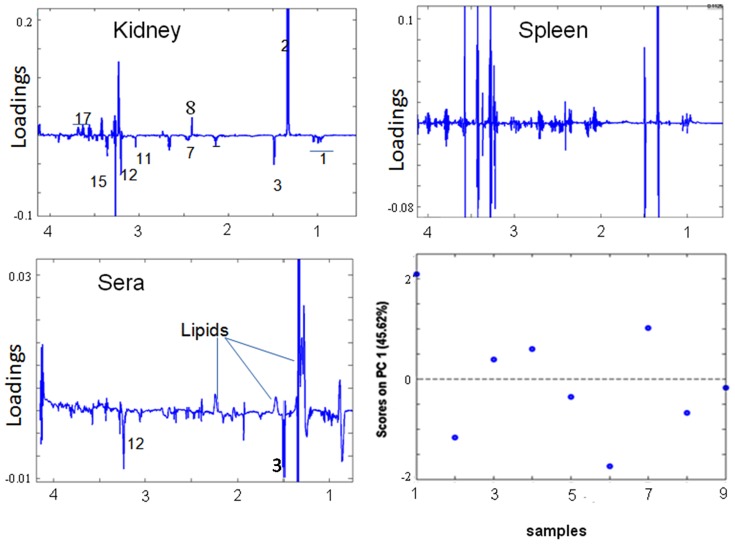
MPCA plot along PC1 of NCM animals. (a) MPCA scores plot. (b–d) MPCA loadings along kidney spleen and serum respectively.

Along PC1, high lipids in the sera are associated to low GPC in serum which accounts for lipid metabolism and unlike in CM (Figure 7). A decrease of glutamine in kidney is associated with increase in lactate, pyruvate, and decrease of alanine and increase of *myo*-inositol in kidney. Glutamine levels in kidney are also negatively related to lipids and lipoproteins in serum and positively with GPC and alanine in serum. It is also associated positively with creatine, BCAA and choline in kidney.

Along PC3 serum lipids are (a) anti-correlated to serum GPC, BCAA. lactate, alanine, glutamine, choline and betaine in kidney, and anti correlated to lactate, alanine, glutamine in spleen. (b) correlated to pyruvate, GPC, glutamate and *scyllo*-inositol in kidney and, BCAA creatine and *scyllo*-inositol in spleen (Figure S4).

#### Control

**Figure 8:**
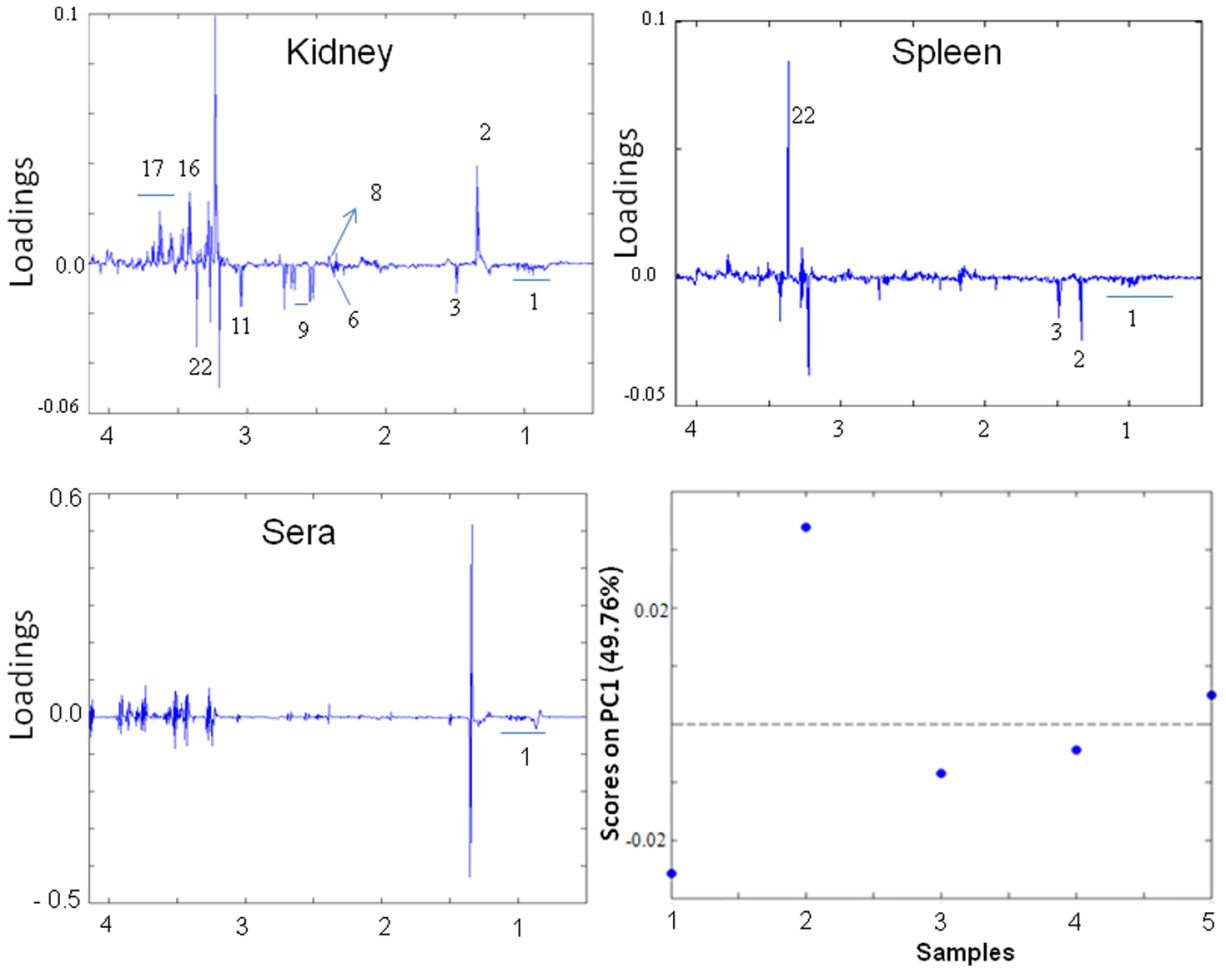
MPCA scores plot along PC1 of control animals. (a) MPCA scores plot. (b–d): MPCA loadings along kidney, spleen and serum respectively.

The numbers of correlations are relatively few as compared to the correlations observed in animals with NCM and CM. Alanine is negatively correlated to lactate, pyruvate, *myo*-inositol and taurine while positively with BCAA, glutamate, *scyllo*-inositol, creatine and citrate in kidney, positively with BCAA in sera and spleen as well as lactate and alanine in spleen (Figure 8). Along PC2 glutamate shows negative relationship with citrate in kidney, alanine and *scyllo*-inositol in spleen and positive association with citrate in spleen (Figure S5). PC3 suggests a positive association of serum glucose with taurine and cholines in spleen. Moreover serum glucose has positive correlation with citrate in kidney and a negative correlation with *myo*-inositol, cholines in kidney and alanine, lactate and *scyllo*-inositol in spleen (Figure S6).

## Discussion

Here we identified distinct metabolic fingerprints of kidney and spleen in mice with NCM, CM and also the functional relationships of metabolites in serum, kidney and spleen in the two classes. Similar to our previous results there are differential perturbations in certain metabolites in CM vis a vis NCM.

Both CM and NCM mice have increased levels of BCAA and alanine in kidney and spleen. The increase in BCAA could be due to their reduced catabolism. Branched chain amino acids are converted to α-ketoacids and further to their Acyl-CoA derivatives. A defect in one or both the pathways will result in accumulation of the same in the tissues. An increase in BCAA is also noticed in liver of both CM and NCM mice in our previous studies. A defect in BCAA metabolism is thus evident in both the infected groups (CM and NCM). Alanine is formed by transamination of pyruvate with glutamine and is subsequently deaminated to liberate ammonia [40]. A possible reason for the increase in alanine could be due to an increased level of ammonia present in kidney for both CM and NCM which results in its formation. The mice afflicted with CM as well as NCM have decreased glutamate in both kidney and spleen and the decrease in case of CM is more with respect to that in NCM as evident from their pairwise OPLS-DA loadings. The carbon units of glutamate enters into TCA cycle, through 2-oxoglutarate, oxaloacetate and posphoenolpyruvate or malate to pyruvate [41]. Betaine is increased in kidney of both CM and NCM. The increase is more in case of CM with respect to NCM. Betaine is an osmolyte present in the kidney medulla and is known to protect cells from extracellular osmolarity [42], [43], [44]. It is also known to stabilize the macromolecules present in the cells by interacting with extracellular tonicity and physiological perturbations [45], [46]. Creatine clearance in known to decrease in acute malaria [47] which could result in its high concentration in kidney. Creatinine, a downstream product of Creatine is also known to have significant lower clearance in acute malaria [48]. This could result in presence of high creatine in kidney of CM mice. A decrease of choline, phosphocholine and GPC is present in both CM and NCM with respect to controls. This could be due to internalization of phosphatidylcholine by the parasites which modifies the turnover of GPC, PC and choline in the host [49]. The functional relationship between the metabolites indicated by MPCA plots shed light on the cross talk among various metabolites. The metabolic correlation across the tissues are different for three groups of animals (CM, NCM and Control) which suggests that the cross talk that happen across the tissues are different in the three groups of animals.

In case of CM mice, glutamate is negatively correlated to alanine and lactate and positively correlated to pyruvate. This correlation points towards a possibility of glutamate entering TCA Cycle, donating its amine group to pyruvate in a transammination reaction. In case of CM mice a decrease of creatine is further associated to increase of glycine and increase of alanine in kidney. This could be because creatine is formed from glycine via guanidoacetate. It is important to note that glycine along with alanine is a part of ammonia recycling pathway. Creatine is further associated positively with cholines in kidney and spleen whereas choline and glycine are negatively correlated. This could be because the down stream product of choline is glycine. Moreover glycine is also positively correlated to lactate in MPCA which points towards the linking of glycolysis to glycine metabolism. Glycolysis is linked to glycine metabolism via serine. Serine is linked to pyruvate by serine dehydratase. Similar correlations are present in spleen of CM as well. The glycolysis in kidney and ammonia recycling are closely linked in case of CM animals. Glycolysis and nitrogen metabolism are thus closely related in CM. The anaerobic condition during malaria could result in the formation of lactate from pyruvate, thus resulting in negative correlation of pyruvate and lactate. An important point to note from MPCA correlations is that along PC2, in CM a low serum glucose is associated with high serum lipids/lipoproteins [2]. This observation also confirms our earlier hypothesis of high serum lipids/lipoproteins in CM being due to the depletion of serum glucose [2]. The further involvement of *o*-phosphoethanolamine with lipids and lipoproteins point to its involvement in lipid metabolism in CM. Our results from PC2 (Supplementary 2b) suggest that glucose is anticorrelated to alanine and lactate in kidney and spleen which accounts for the glycolysis. Moreover a decrease in glucose in sera is associated to a decrease in *myo*-inositol in kidney which could be because *myo*-inositol is derived from glucose. Further creatine is metabolically linked to urea cycle [50] and glycerophospholipid [51] metabolism. It is formed from serine which in turn is sourced from pyruvate. Our observation of negative correlation of creatine in kidney of mice with CM with serum glucose, lipids/lipoproteins, GPC and choline and a positive correlation with lactate and alanine in kidney suggests that an increase in creatine could actually be driven by enhanced glycolysis and breakdown of glycerophospholids. In case of CM there is the presence of negative correlation of glutamate in kidney and lactate in serum (PC 3) unlike in NCM. During fatal stage of CM the glutamate in kidney forms glucose which in turn forms lactate. Moreover the cross talk among choline, *myo*-inositol, *scyllo*-inositol and glycine in various tissues indicates their specific roles in maintaining osmotic pressure and transmethylation reaction [52], [53] that occur in these organs. The level of m*yo-*inositol and *scyllo*-inositol in kidney and spleen is low in mice with NCM with respect to CM. *Myo*-inositol is converted to glucuronic acid [54] which in turn is a substrate for detoxification. Moreover it is also involved in glycerphospholipid formation [55]. A negative correlation of *myo*-inositol of kidney with serum GPC in NCM mice is indicative of this fact. *Scyllo*-inositol, is produced from *myo*-inositol by isomerization via *myo*-innose [56]. The evolution the levels of *scyllo*-inositol and myo-inositol are parallel in normal un-infected animals and in some of the pathological conditions [57]. In NCM a decrease of *myo*-inositol could affect the formation of *scyllo*-inositol in spleen. Further *o*-phosphoethanolamine has an important role in NCM animals. *O*-phosphoethanolamine is an intermediate of phospholipid metabolism and also a building block for phospholipid synthesis. Here we observe that a decrease in *o*-phosphoethanolamine of spleen is associated with a decrease in serum lipids which in turn is linked to an increase in serum choline and GPC. A striking difference is noted when MPCA of CM, NCM and control are compare to each other. The functional relationships across the tissues are different in the three groups of animals. In other words the dominant events reflected by PC1 of MPCA are different in CM, NCM and control. In case of control the only metabolites in serum which is involved in cross talk with kidney and spleen is BCAA however in case of NCM lipoproteins, BCAA, alanine and GPC are involved in maintaining homeostasis while in case of CM mice, BCAA, glutamine, acetate and creatine are involved in maintaining homeostasis. This indicates that the differential involvement of serum parameters in two different diseased state. Another striking result of the MPCA plot is that in case of NCM mice spleen has no dominant metabolic correlation with serum and kidney which is not the case with CM and control. However the mode of these correlations is different in CM with respect to controls.

From this study it is evident that the kidney dysfunction and splenomegaly causes differential perturbation of metabolism in CM and NCM. In CM host, one of the possible reasons of the kidney dysfunction could be the increase in the extracellular osmolarity reflected by the increase of betaine. A glutamate decrease in kidney and spleen in CM could be due a perturbation in the glutamate transportation. This is because the glutamate transport is driven by pH gradient and is accompanied by intracellular acidification [58]. Under metabolic acidosis in cerebral malaria [59] this phenomenon is likely to be perturbed. Metabolic acidosis could also deactivate phosphate independent glutaminase (PIG) [60] thereby decreasing the production of glutamate and also reduced glutamate uptake by the glutamate transporter EAAC1 [61]. Metabolic acidosis also accelerates glutamate flux through glutamate dehydrogenase [62] thereby further reducing the glutamate concentration. Furthermore, phosphate dependent glutaminase (PDH) is activated under these circumstances [63]. The role of acidic amino acid in the nephron is the cell volume regulation [64]. Under the hypertonic stress condition, there is increased expression of glutamate transporters EACC1 [65]. Thus a decrease of glutamate in CM can cause a kidney dysfunction by affecting the cell volume. In addition, ‘dicarboxylic aminoaciduria’, a disease in which the glutamate filtration rate exceeds glomerular filtration, is known to cause ‘seizures’ [66], a common symptom of CM.

## Conclusion

The metabolic fingerprint of spleen and kidney are very distinct in CM with respect to that in NCM and controls. It is evident that kidney dysfunction and splenomegaly in cerebral malaria has an effect in host metabolism. It is evident from this study that there are differential perturbation in the levels of certain metabolites in kidney and spleen in CM with respect to NCM and controls. Decreased levels of glutamate in spleen and kidney and increased levels of creatine in kidney are some of the fingerprints of CM. The cross talk between various metabolites across tissues in CM, NCM and Control are different. Correlation studies in conjunction with previously reported results point towards a perturbation in lipid metabolism, TCA cycle, ammonia detoxification pathway, and glucose to *myo*-inositol conversion in cerebral malaria.

## Supporting Information

Figure S1 **OPLS-DA scores plot of ^1^H NMR spectra of kidney and spleen of CM, NCM and Control and ROC plot of the cross validated Y of the OPLS-DA model of CM and NCM.** (a) OPLS-DA scores plot of kidney. (b) ROC plot of kidney of CM and NCM. (c) OPLS-DA scores plot of spleen. (d) ROC plot of spleen. The red, black and blue symbols represent CM, NCM and Control animals respectively.
(TIF)

Figure S2 **MPCA plot along PC2 of CM animals.** (a–c): MPCA loadings along kidney spleen and serum respectively. (d) MPCA scores plot.
(TIF)

Figure S3 **MPCA plot along PC3 of CM animals.** (a–c): MPCA loadings along kidney spleen and serum respectively. (d) MPCA scores plot.
(TIF)

Figure S4 **MPCA plot along PC3 of NCM animals.** (a–c): MPCA loadings along kidney spleen and serum respectively. (d) MPCA scores plot.
(TIF)

Figure S5 **MPCA plot along PC2 of control animals.** (a–c): MPCA loadings along kidney spleen and serum respectively. (d) MPCA scores plot.
(TIF)

Figure S6 **MPCA plot along PC3 of control animals.** (a–c): MPCA loadings along kidney spleen and serum respectively. (d) MPCA scores plot.
(TIF)

Table S1 **Concentration in (mmol/Kg) of the metabolites in kidney and spleen.**
(DOCX)
