# Supplementary figures and images for: Metabolic Perturbations of Kidney and Spleen in Murine Cerebral Malaria: 1H NMR-Based Metabolomic Study
Source: PLoS One. 2013 Sep 6;8(9):e73113. doi: 10.1371/journal.pone.0073113 (PMC3765208; doi:10.1371/journal.pone.0073113)

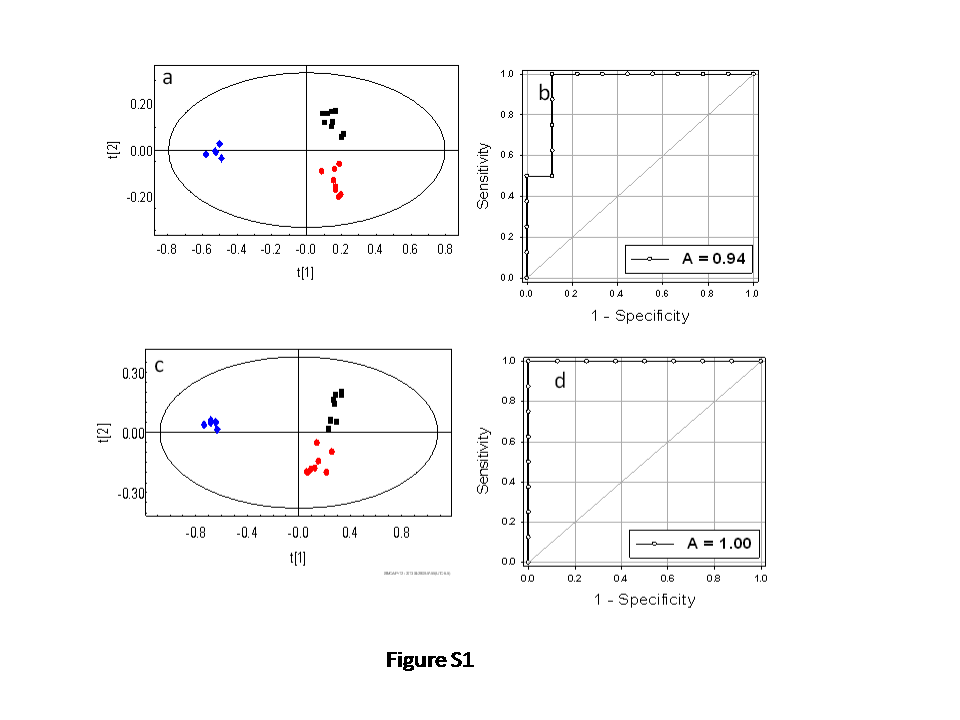

Supplement: Figure S1 — OPLS-DA scores plot of 1H NMR spectra of kidney and spleen of CM, NCM and Control and ROC plot of the cross validated Y of the OPLS-DA model of CM and NCM. (a) OPLS-DA scores plot of kidney. (b) ROC plot of kidney of CM and NCM. (c) OPLS-DA scores plot of spleen. (d) ROC plot of spleen. The red, black and blue symbols represent CM, NCM and Control animals respectively. (TIF) [file pone.0073113.s001.tif]

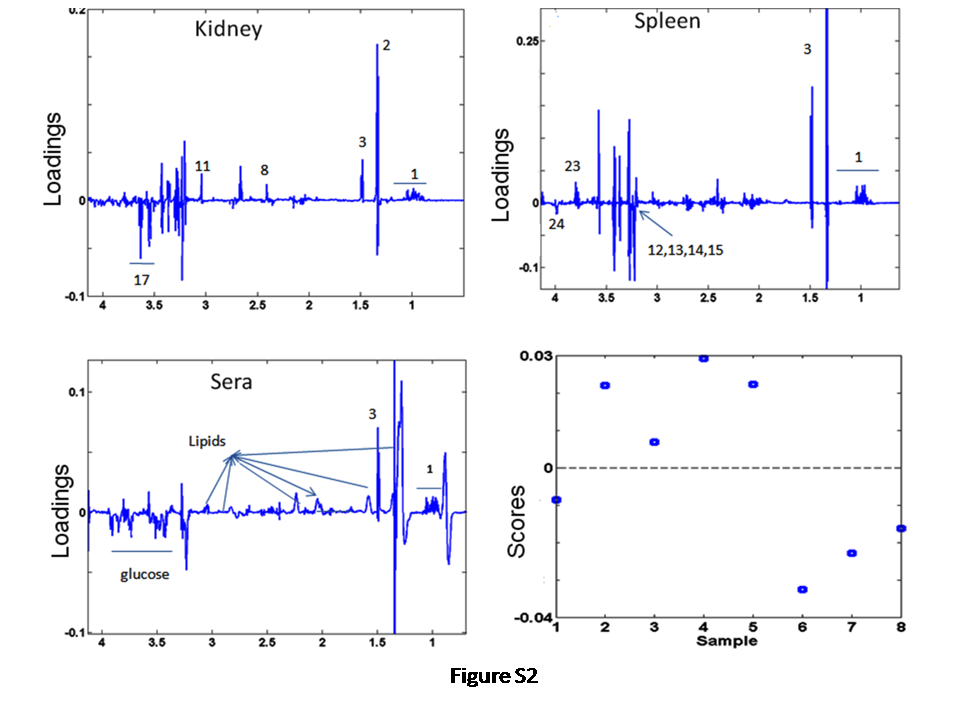

Supplement: Figure S2 — MPCA plot along PC2 of CM animals. (a–c): MPCA loadings along kidney spleen and serum respectively. (d) MPCA scores plot. (TIF) [file pone.0073113.s002.tif]

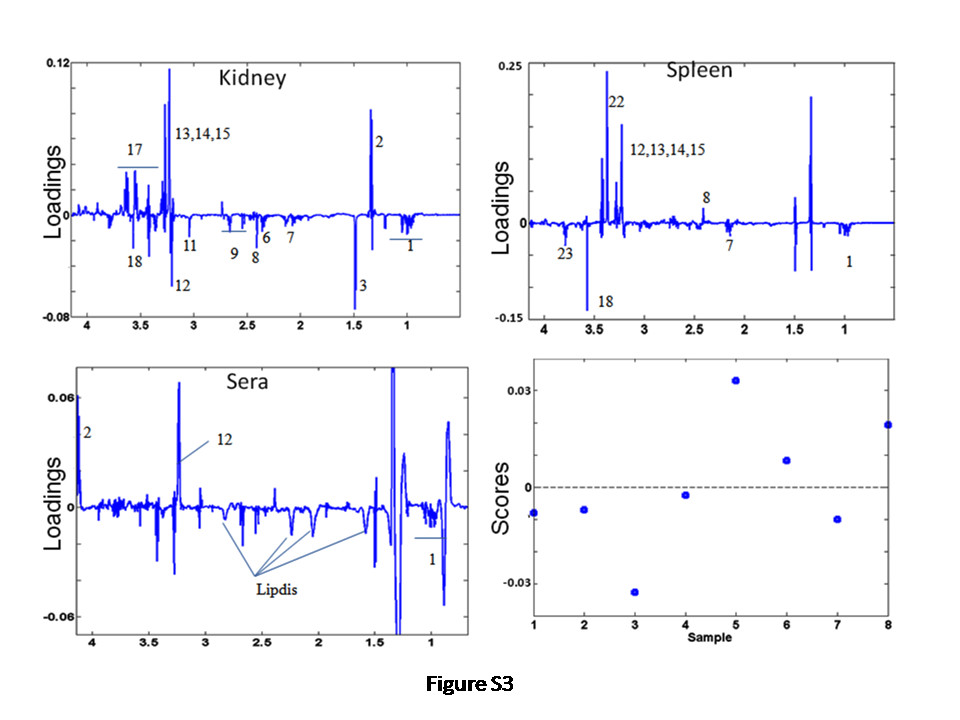

Supplement: Figure S3 — MPCA plot along PC3 of CM animals. (a–c): MPCA loadings along kidney spleen and serum respectively. (d) MPCA scores plot. (TIF) [file pone.0073113.s003.tif]

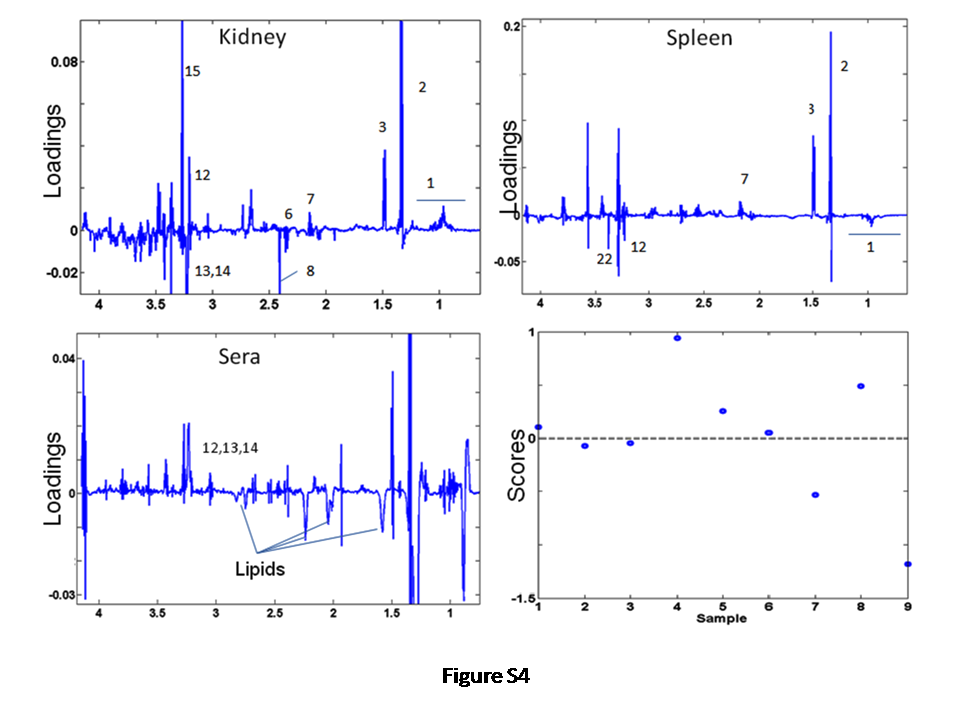

Supplement: Figure S4 — MPCA plot along PC3 of NCM animals. (a–c): MPCA loadings along kidney spleen and serum respectively. (d) MPCA scores plot. (TIF) [file pone.0073113.s004.tif]

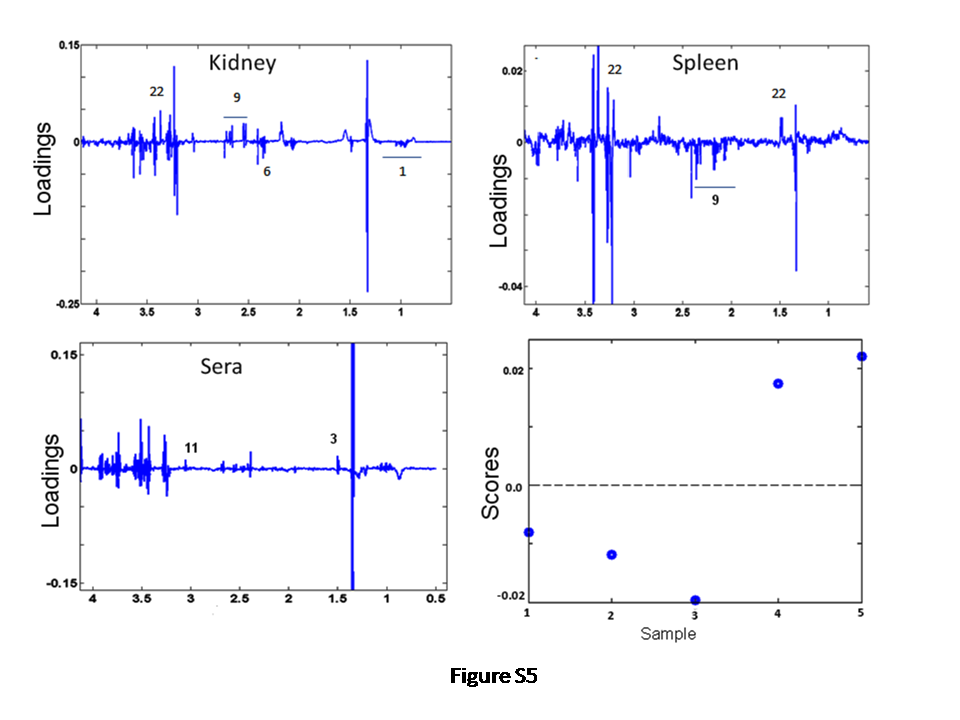

Supplement: Figure S5 — MPCA plot along PC2 of control animals. (a–c): MPCA loadings along kidney spleen and serum respectively. (d) MPCA scores plot. (TIF) [file pone.0073113.s005.tif]

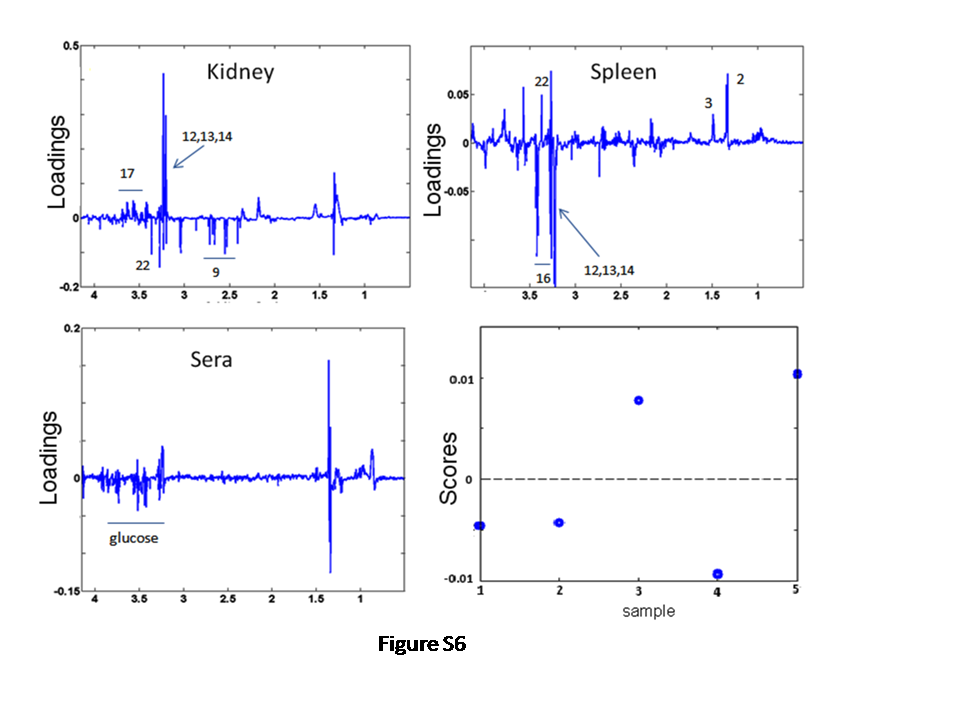

Supplement: Figure S6 — MPCA plot along PC3 of control animals. (a–c): MPCA loadings along kidney spleen and serum respectively. (d) MPCA scores plot. (TIF) [file pone.0073113.s006.tif]
