# Supplementary material for: Metabolic Perturbations of Kidney and Spleen in Murine Cerebral Malaria: 1H NMR-Based Metabolomic Study
Source: PLoS One. 2013 Sep 6;8(9):e73113. doi: 10.1371/journal.pone.0073113 (PMC3765208; doi:10.1371/journal.pone.0073113)
Supplement: Table S1 — Concentration in (mmol/Kg) of the metabolites in kidney and spleen. (DOCX) [file pone.0073113.s007.docx]

Table S1. Concentration in mmol/Kg of the significant metabolites in spleen and kidney of control, CM and NCM.

| Tissue | Metabolite | Control | NCM | CM |
| --- | --- | --- | --- | --- |
| Kidney | Glutamate | 5.67±0.18 | 4.44±0.17 | 3.37±0.24 |
|  | Betaine | 0.21±0.01 | 0.45±0.03 | 0.57±0.03 |
| Spleen | Glutamate | 4.01±0.15 | 3.07±0.27 | 2.03±0.23 |
